# Extracellular Yellow Pigments Produced by *Chaetomium globosum* H-1 Isolated from Spalted Wood: Fermentation Regulation, Multidimensional Characterization, and Sustainable Wool Dyeing

**DOI:** 10.3390/microorganisms14081818

**Published:** 2026-08-18

**Authors:** Boxi Chen, Mengqi Wu, Jianping Sun

**Affiliations:** College of Civil Engineering and Architecture, Guangxi University, Nanning 530004, China

**Keywords:** *Chaetomium globosum*, extracellular yellow pigment, stepwise fermentation screening, functional characterization, wool dyeing

## Abstract

Microbial extracellular pigments are renewable colorants for sustainable textile dyeing. A yellow-pigment-producing fungal strain, H-1, isolated from spalted wood, was identified as *Chaetomium globosum* and evaluated for pigment production, characterization, stability, bioactivity, and wool dyeing. Stepwise single-factor screening indicated suitable culture conditions of initial pH 7, 25 °C, 10 d, 25 g/L glucose, 7 g/L fish peptone, and 0.25 g/L K_2_HPO_4_. Fourier-transform infrared spectroscopy (FTIR) revealed hydroxyl, aromatic, and C-O-C groups, and ultra-high-performance liquid chromatography-high-resolution tandem mass spectrometry (UHPLC-HRMS/MS) putatively annotated representative components including *Chaetomugilin D* and *Armochaetoglobin G*. The crude pigment produced preliminary inhibition zones of 14.30 ± 0.04 and 10.01 ± 0.17 mm against Staphylococcus aureus and Escherichia coli, respectively, while crude fermentation filtrates showed measurable hydroxyl and 2,2-diphenyl-1-picrylhydrazyl (DPPH) radical-scavenging capacities under fixed assay conditions. The pigment was more stable under neutral to alkaline pH and short-term heating at 40–100 °C. Wool showed higher dye affinity than cotton, linen, and silk. Response surface methodology (RSM) optimized wool dyeing at pH 2.35, 91 °C, 80 min, and 2.04 g/L potassium aluminum sulfate, yielding a color strength (K/S) value of 11.65. After three dye-bath reuse cycles, K/S decreased by 16.28%, and washing/rubbing fastness remained above grade 3 under GB/T tests. These results support further development of *Chaetomium globosum* H-1 extracellular pigments for natural wool coloration and dye-bath reuse.

## 1. Introduction

Color is a critical quality attribute in foods, textiles, cosmetics, medicine, and functional materials, influencing not only product appearance and consumer perception but also product identification, quality evaluation, and functional expression [1,2]. Conventional synthetic dyes offer broad color ranges, high tinting strength, and mature processing technologies; however, their production relies heavily on petrochemical feedstocks, and dyeing processes are often associated with high water consumption, energy demand, and colored wastewater discharge [3]. With the rapid development of green manufacturing, low-carbon technologies, and bio-based materials, natural pigments derived from renewable resources have become an important research direction in colorant development [4].

Natural pigments are mainly obtained from plants, animals, and microorganisms. Plant pigments are abundant, but their raw-material supply, pigment content, and quality are readily affected by cultivar, season, geographic origin, and climatic conditions; animal-derived pigments have longer production cycles and limitations in scalable resource utilization. Microorganisms offer short growth cycles, controllable cultivation conditions, broad substrate adaptability, and compatibility with continuous production, enabling scalable pigment preparation through liquid or solid-state fermentation. By regulating carbon sources, nitrogen sources, inorganic salts, pH, and temperature, microbial growth, metabolic flux distribution, and extracellular pigment accumulation can be directed, providing a robust process foundation for efficient pigment production [5,6].

Filamentous fungi are major sources of microbial pigments, and their metabolic systems can generate diverse colored compounds, including carotenoids, quinones, polyketides, azaphilones, and cytochalasans [7,8,9]. Compared with intracellular pigments, extracellular pigments are released directly into the culture broth and can be separated from fungal biomass by centrifugation and filtration, thereby simplifying downstream processing [10,11,12]. Fungal pigments commonly contain hydroxyl, carbonyl, aromatic, and conjugated structures, which not only determine their light-absorption and chromatic behavior but may also endow them with antibacterial, radical-scavenging, and metal-coordination functions. These features allow fungal pigments to act simultaneously as colorants and functional components [13,14,15].

*Chaetomium globosum* is a widely distributed filamentous fungus with strong environmental adaptability and rich secondary-metabolic potential. Its metabolites include chaetoglobosins, azaphilones, chaetomugilins, armochaetoglobins, and other structurally diverse compounds. Different strains and cultivation conditions can generate extracellular metabolites with distinct compositions and colors. Therefore, integrating taxonomic identification, cultivation-condition screening, and spectroscopic/mass-spectrometric characterization is valuable for systematically understanding *C. globosum*-derived pigments from the perspectives of biological properties, production efficiency, and material composition [16,17,18,19].

Textile dyeing provides a representative high-value application scenario for microbial pigments. Wool is a protein fiber rich in amino, carboxyl, and amide groups, which can interact with oxygen- and nitrogen-containing pigment molecules through electrostatic attraction, hydrogen bonding, and coordination under acidic dye-bath and thermal-treatment conditions [20,21,22,23]. Mordants can further construct pigment-metal ion-fiber bridging structures, thereby improving dye adsorption and fixation efficiency. Meanwhile, regulating dye-bath pH, temperature, time, and mordant dosage, together with dye-bath recycling, can improve pigment and mordant utilization while maintaining high color depth, highlighting the resource-efficient advantages of natural dyeing processes [24,25,26].

Although fungal pigments have attracted increasing attention as renewable colorants, extracellular yellow pigments produced by *Chaetomium globosum*, particularly strains isolated from spalted wood, remain insufficiently characterized. Previous studies on fungal pigments have often focused on pigment production, chemical profiling, biological activity, or textile dyeing separately, while limited work has integrated strain identification, cultivation-condition screening, pigment characterization, processing stability, preliminary bioactivity, dyeing performance, and dye-bath reuse into a single application-oriented framework. This gap limits the systematic evaluation of *C. globosum*-derived extracellular pigments as potential natural textile colorants. To address this gap, the present study isolated a stable yellow-pigment-producing fungal strain, H-1, from spalted wood and identified it as *C. globosum* through morphological observation and ITS sequence analysis. Cultivation time, initial pH, temperature, carbon source, nitrogen source, and inorganic salt composition were screened using dry cell weight (DCW) and optical density at 273 nm (OD_273_) as evaluation indices. FTIR and UHPLC-HRMS/MS further investigated the crude extracellular pigment for structural features and representative putatively annotated components, and its processing stability, preliminary antibacterial effects, and antioxidant-related characteristics during fermentation were evaluated. In addition, wool dyeing performance was systematically assessed through fabric comparison, single-factor dyeing experiments, Box–Behnken response-surface design, and dye-bath reuse. The novelty of this work lies in integrating spalted-wood-derived *C. globosum* strain resources, extracellular pigment production, multidimensional pigment characterization, and sustainable wool dyeing evaluation into a unified workflow for the development of microbial natural colorants.

## 2. Materials and Methods

### 2.1. Materials and Microorganisms

Strain H-1 was isolated from spalted wood collected in Guangxi, China. Potato dextrose agar (PDA) and potato dextrose broth (PDB) for cultivation were purchased from Guangdong Huankai Microbial Technology Co., Ltd. (Guangzhou, China) Glucose, fructose, sucrose, maltose, starch, fish peptone, yeast extract, urea, NH_4_Cl, KCl, KH_2_PO_4_, K_2_HPO_4_, MgCl_2_, HCl, NaOH, H_2_O_2_, Na_2_SO_3_, gallic acid, rutin, NaNO_2_, Al(NO_3_)_3_, FeSO_4_, salicylic acid, and potassium aluminum sulfate were of analytical or biochemical reagent grade. Folin–Ciocalteu reagent and DPPH solution were used for total phenol and radical-scavenging assays. *Staphylococcus aureus* and *Escherichia coli* were used as antibacterial indicator strains. These two bacterial strains were laboratory-maintained stock cultures used for routine antibacterial screening in our laboratory and were not newly isolated or deposited strains in the present study; therefore, no accession numbers were assigned. Cotton, linen, silk, and wool fabrics were used as dyeing substrates.

### 2.2. Isolation, Purification, and Identification of Strain H-1

The pigment-producing strain was isolated from spalted wood using a surface-sterilized wood-fragment plating approach modified from a previously reported method for isolating and screening fungi from spalted wood [27]. Wood pieces containing zone lines were cut into blocks of approximately 2 cm × 1 cm × 1 cm, immersed in 75% ethanol for 10–15 s, washed in sterile water for 5 min, rinsed three times, and dried with sterile filter paper. The treated wood blocks were placed on PDA plates, with 1–2 pieces per plate, and incubated in the dark at 25 ± 1 °C for 7 d. Colonies showing vigorous growth and yellow coloration around the wood blocks were selected. A sterile punch was used to excise 6 mm mycelial plugs from the colony margin, which were transferred to the center of fresh PDA plates. After consecutive subculturing until a uniform colony morphology was obtained, the purified strain was inoculated onto PDA slants, incubated in the dark at 25 ± 1 °C for 5 d, and stored at 4 °C.

Morphological identification was performed on PDA plates. The strain was incubated in the dark at 25 ± 1 °C, and colony front and reverse morphology, color, texture, and pigment-producing characteristics were recorded continuously. Small amounts of mycelia and ascospores were mounted in water and observed under 40× and 100× objectives to examine septa in appendage hairs and spore morphology. Molecular identification was performed by amplifying the fungal ITS region using ITS1/ITS4-R primers. The PCR reaction volume was 25 μL, and the amplification program was 95 °C for 5 min; 30 cycles of 94 °C for 30 s, 57 °C for 30 s, and 72 °C for 90 s; followed by 72 °C for 10 min. The amplified product was confirmed by 1.5% agarose gel electrophoresis and sequenced. The obtained sequence was compared with sequences in the NCBI database using the NCBI BLASTn online service, and a neighbor-joining phylogenetic tree was constructed in MEGA 11.0. The ITS sequence of H-1 was deposited under accession number PQ573295.

### 2.3. Stepwise Fermentation Screening and Pigment Quantification

The basal liquid medium contained 20 g/L glucose and 5 g/L yeast extract. The medium was dispensed at 30 mL into 100 mL culture vessels, sterilized at 121 °C for 20 min, cooled, inoculated with one 6 mm mycelial plug, and statically incubated in the dark to minimize uncontrolled light exposure and possible light-induced changes in pigment production or pigment stability. For growth-curve analysis, the strain was cultivated in PDB at 25 ± 1 °C for 12 d, and three culture bottles were randomly sampled every 24 h to determine mycelial dry weight and pigment absorbance.

The effects of cultivation conditions were examined using initial pH values of 4, 5, 6, 7, 8, 9, and 10 and cultivation temperatures of 20, 25, and 30 °C. For medium-composition screening, glucose, fructose, sucrose, maltose, and starch were individually tested as 20 g/L carbon sources, and glucose concentrations of 10, 15, 20, 25, and 30 g/L were further evaluated. Fish peptone, yeast extract, urea, and NH_4_Cl were individually tested as 5 g/L nitrogen sources, and fish peptone concentrations of 1, 3, 5, and 7 g/L were further evaluated. KCl, KH_2_PO_4_, K_2_HPO_4_, and MgCl_2_ were separately added at 0.5 g/L, and K_2_HPO_4_ concentrations of 0.25, 0.5, 1, 3, and 5 g/L were further tested. Each group was cultivated for 10 d with five replicates per condition. This fermentation experiment was designed as a stepwise single-factor screening to identify suitable cultivation ranges and medium components, rather than as a multifactorial design for modeling interaction effects. Each treatment consisted of five independently inoculated culture vessels (n = 5), whereas the growth-curve experiment used three independently sampled culture vessels per time point (n = 3).

After cultivation, the fermentation broth together with the mycelial mat was transferred to centrifuge tubes and centrifuged at 6000 r/min for 5 min. The mycelia were washed with deionized water and dried at 60 °C to constant weight; dry cell weight (DCW) was calculated from the mass difference before and after drying. The supernatant was filtered through a 0.22 μm membrane, and absorbance at 273 nm was measured as a relative index of extracellular pigment-containing chromophoric products, expressed as OD_273_. In the initial-pH experiment, the difference between OD_273_ after and before cultivation (ΔOD_273_) was used to eliminate background absorbance differences among media.

### 2.4. Preparation of Extracellular Pigment Samples

The strain was cultivated for 10 d under the selected cultivation conditions. The fermentation broth was centrifuged at 6000 r/min for 5 min and filtered through a 0.22 μm membrane to obtain a clarified extracellular yellow pigment solution. Part of the filtrate was frozen at −80 °C for 12 h and lyophilized to prepare solid pigment samples. Lyophilized pigment was dissolved in water at different mass concentrations to establish a calibration curve between concentration and OD_273_. To justify the monitoring wavelength, the clarified extracellular pigment solution and the lyophilized pigment redissolved in deionized water were scanned over 200–800 nm using deionized water or the corresponding uninoculated medium as the blank. The pigment preparation showed its strongest and most reproducible absorption band at 273 nm, together with a gradually decreasing absorption tail in the visible region of 350–600 nm, which was consistent with the observed yellow-brown color. Because no sharp visible-region maximum was observed, OD_273_ was used as a relative index for extracellular pigment-containing chromophoric products rather than as a selective quantification of a single purified yellow compound. The concentration calculated from this calibration curve represents a crude lyophilized pigment-equivalent concentration of the extracellular pigment preparation. Because the pigment preparation was not purified into individual compounds, chromatographic purity and the yield of a single pigment molecule were not determined in this study. The pigment solution used for dyeing was uniformly diluted with deionized water to OD_273_ = 1.064 ± 0.02, corresponding to a crude lyophilized pigment-equivalent concentration of 0.5 g/L.

### 2.5. Characterization

FTIR analysis was performed on a Nicolet iS50 Fourier-transform infrared spectrometer (Thermo Fisher Scientific, Waltham, MA, USA) using the KBr pellet method. Approximately 1 mg of lyophilized pigment was thoroughly ground with 100 mg of dried KBr and pressed into a transparent pellet. Spectra were collected against an air background over the range of 4000–400 cm^−1^ with 32 scans and a resolution of 4 cm^−1^. UHPLC-HRMS/MS analysis was performed using an UltiMate 3000 UHPLC system coupled to a Q Exactive mass spectrometer (Thermo Fisher Scientific, Waltham, MA, USA) equipped with an Atlantis T3 column (100 mm × 3.0 mm, 3.0 μm; Waters Corporation, Milford, MA, USA). The column temperature was 30 °C, the injection volume was 10 μL, and the flow rate was 0.4 mL/min. Mobile phase A was 0.1% formic acid in water, and mobile phase B was acetonitrile. The gradient was as follows: 0–1 min, 50% B; 1–16 min, 50–100% B; 16–17 min, returned to 50% B; and 17–20 min, maintained at 50% B. For the HESI source, sheath gas and auxiliary gas were set to 40 and 10 arb, respectively; spray voltages were 3.8 kV in positive mode and 3.0 kV in negative mode; capillary temperature was 320 °C; and auxiliary gas temperature was 300 °C. Data were acquired in Full MS/dd-MS^2^ top 10 mode, with a full-scan resolution of 70,000 over m/z 100–1000 and an MS^2^ resolution of 17,500. Components were annotated according to retention time, accurate molecular ions, isotope distribution, and MS^2^ fragment characteristics. Because authentic standards and nuclear magnetic resonance (NMR) data were not available, the UHPLC-HRMS/MS results were used for putative annotation rather than definitive structural identification. The pigment preparation was analyzed as a crude extracellular mixture, and chromatographic purity of individual components was not determined.

### 2.6. Stability Evaluation

For the stability evaluation, pigment residual rate (%) was not calculated from pigment dry weight or normalized to fungal biomass. Instead, it was calculated from the ratio of OD_273_ before and after treatment: residual rate (%) = A_t_/A_0_ × 100, where A_0_ and A_t_ represent the absorbance of the extracellular pigment solution before and after treatment, respectively. Thus, the percentage values reported for the stability evaluation indicate OD_273_ retention of the pigment-containing solution. When pigment concentration is expressed in this study, it refers to crude lyophilized pigment-equivalent concentration on a *w*/*v* basis (g/L solution), rather than % *w*/*w* of biomass. For pH stability, 5 mL of pigment solution was adjusted to pH 2, 4, 6, 8, or 10 and treated in the dark at room temperature for 8 h, with measurements every 2 h. For thermal stability, samples were treated in the dark at 40, 60, 80, and 100 °C for 2 h, with measurements every 0.5 h. For oxidative stability, 1 mL of 0.10%, 0.25%, or 0.50% H_2_O_2_ was added to 5 mL of pigment solution. For reductive stability, 1 mL of Na_2_SO_3_ at 1, 2.5, 5, 25, or 50 μg/mL was added. Both treatments were conducted in the dark for 6 h, with measurements every 2 h. For UV stability, 5 mL of pigment solution was placed 15 cm from a 30 W UV lamp and irradiated for 8 h, with measurements every 2 h. For metal-ion stability, 1 mL of 0.1 mol/L metal-ion solution (K^+^, Na^+^, Li^+^, Mg^2+^, Ca^2+^, Mn^2+^, Zn^2+^, Cu^2+^, Pb^2+^, Fe^3+^, or Al^3+^) was added to 5 mL of pigment solution and kept in the dark at room temperature for 2 h. OD_273_ values before and after treatment were measured, and visual color changes and precipitation were recorded. All treatments were performed in triplicate.

### 2.7. Antibacterial Activity and Antioxidant-Related Assays

Antibacterial activity was assessed using the agar well diffusion method. Suspensions of *S. aureus* and *E. coli* were adjusted to 1 × 10^7^ CFU/mL with PBS, and 100 μL of each suspension was evenly spread onto Mueller-Hinton agar plates. Two wells with a diameter of 6 mm were prepared on each plate and filled with 50 μL of pigment solution or an equal volume of sterile deionized water. Sterile deionized water was used as the negative control because it was the solvent used to dissolve and dilute the pigment preparation, and therefore allowed evaluation of any background effect from the solvent itself. No antibiotic positive control was included in the original experimental design, because the agar-well diffusion assay was intended as a preliminary screening of inhibitory potential rather than a quantitative antimicrobial comparison. This limitation is further discussed in Section 3.3. After incubation in the dark at 37 °C for 24 h, inhibition-zone diameters were measured using the cross-measurement method.

Three fermentation filtrate samples were collected every 24 h for 10 d to determine pH, reducing sugar, total phenol, and flavonoid contents. For reducing sugar determination, 0.5 mL of sample, 1.5 mL of water, and 1.5 mL of DNS reagent were mixed, heated in a boiling water bath for 5 min, cooled, diluted to 10 mL, and measured at 540 nm; concentrations were calculated using a glucose calibration curve. Total phenols were determined using the Folin–Ciocalteu method: 0.5 mL of sample was mixed with 2 mL of water and 0.5 mL of 1 mol/L Folin–Ciocalteu reagent for 5 min, followed by the addition of 3 mL of 7% Na_2_CO_3_. The mixture was reacted at 40 °C for 30 min, cooled, and measured at 760 nm, with results expressed as gallic acid equivalents. Flavonoids were determined using the NaNO_2_-Al(NO_3_)_3_ colorimetric method with rutin as the standard and absorbance measured at 510 nm.

For the hydroxyl radical-scavenging assay, 1 mL of pigment solution was sequentially mixed with 1 mL of 3 mmol/L FeSO_4_, 1 mL of 3 mmol/L salicylic acid-ethanol solution, and 0.2 mL of 0.3% H_2_O_2_; the mixture was reacted at 37 °C for 30 min and measured at 510 nm. For the DPPH radical-scavenging assay, 3 mL of 0.2 mmol/L DPPH solution was mixed with 1 mL of pigment solution and reacted in the dark at room temperature for 30 min, followed by measurement at 517 nm. Radical-scavenging activity was calculated as [1 − (A_1_ − A_2_)/A_0_] × 100%, where A_1_ is the absorbance of the sample reaction system, A_0_ is the absorbance of the blank system, and A_2_ is the background absorbance of the sample. These assays were conducted using fermentation filtrates collected at different cultivation times under a fixed sample-to-reagent ratio, with the purpose of monitoring temporal changes in radical-scavenging activity during fermentation. The original experimental design did not include a concentration-response series of crude or purified pigment samples, nor did it include a Trolox, ascorbic acid, or other standard antioxidant calibration curve. Therefore, half-maximal inhibitory concentration (IC50) values and Trolox-equivalent antioxidant capacity could not be calculated from the available data.

### 2.8. Fabric Screening, Wool Dyeing, Color Measurement, and Dye-Bath Reuse

For preliminary fabric screening, 1 g each of cotton, linen, silk, and wool was immersed in 0.5 g/L pigment solution at a liquor ratio of 1:20 and dyed at 80 °C for 60 min. After dyeing, the fabrics were rinsed with deionized water and air-dried. This screening step was performed to compare the affinity of the extracellular pigment toward different fabric substrates, and wool was selected for the subsequent dyeing-parameter experiments, RSM optimization, and dye-bath reuse tests because it showed the best dyeing performance among the tested fabrics. Before single-factor wool dyeing experiments, wool fabrics (10 cm × 15 cm) were pretreated at a liquor ratio of 1:30 in 1 g/L neutral soap solution at room temperature for 10 min, washed, and dried. The effects of dye-bath pH (1–7), dyeing temperature (60–100 °C), dyeing time (50–90 min), and potassium aluminum sulfate concentration (0.5–2.5 g/L) were investigated. Unless otherwise specified, the baseline conditions were pH 4, 80 °C, 60 min, and a liquor ratio of 1:20. Unless otherwise stated, the same 0.5 g/L pigment solution was used for the single-factor dyeing experiments, RSM validation, and dye-bath reuse experiments. Potassium aluminum sulfate was added via simultaneous mordanting, and each treatment was performed in triplicate.

Fabrics were folded into two layers, and L*, a*, b*, and reflectance R at the maximum absorption wavelength were measured using a CS-600 spectrophotometer under D65 illumination and a 10° observer angle. Each sample was measured four times, and the mean value was used. Total color difference was calculated as ΔE = [(ΔL*)^2^ + (Δa*)^2^ + (Δb*)^2^]^1/2^, and color strength was calculated using the Kubelka-Munk equation K/S = (1 − R)^2^/(2R). Rubbing fastness and washing fastness were determined according to GB/T 3920-2008 [28] and GB/T 3921-2008 [29], respectively. For rubbing fastness, dry and wet rubbing tests were performed and rated using the standard gray scale. For washing fastness, dyed wool samples were washed with a standard soap solution under the GB/T 3921-2008 procedure, and color change and staining on adjacent cotton and wool fabrics were evaluated using the gray scale. The fastness ratings were expressed on a grade scale from 1 to 5, where grade 5 indicates the best fastness. Lightfastness was not evaluated in the original experimental design.

Dye-bath reuse was performed under the response-surface validation conditions, namely pH 2.35, 91 °C, 80 min, and 2.04 g/L potassium aluminum sulfate. After each dyeing cycle, the wool fabric was removed, and deionized water was added to the remaining dye bath to restore the liquor ratio to 1:20 without supplementing pigment or mordant. Because the volume of water added after each cycle was not recorded separately, the measured OD_273_ and Al^3+^ values in the reused dye baths should be interpreted as apparent residual concentrations after reuse, which may include both component removal by fiber adsorption/complexation/precipitation and dilution caused by water compensation. The bath was reused for five consecutive cycles, designated I–V. The chromatic parameters, K/S values, and color fastness of the dyed fabrics were measured after each cycle. The OD_273_ and Al^3+^ concentrations of the post-dyeing bath were also determined, with Al^3+^ quantified using an iCAP PRO system.

### 2.9. Response Surface Design and Data Analysis

Response surface methodology (RSM) was conducted using a four-factor, three-level Box–Behnken design (BBD) constructed in Design-Expert 13, with color strength (K/S) as the response value. The design consisted of 27 experimental runs, including three replicated center points (runs 10, 26, and 27), which were used to estimate the pure error and evaluate the lack of fit. The factors were dye-bath pH (A: 1, 2, and 3), dyeing temperature (B: 80, 90, and 100 °C), dyeing time (C: 70, 80, and 90 min), and potassium aluminum sulfate concentration (D: 1.5, 2.0, and 2.5 g/L). A quadratic polynomial regression model was established, and analysis of variance was used to evaluate model significance, lack of fit, and coefficients of determination. Unless otherwise stated, data are presented as mean ± standard deviation (SD). For the stepwise fermentation-screening experiments, each treatment consisted of independently inoculated culture vessels (n = 5), whereas the growth-curve experiment used independently sampled culture vessels at each time point (n = 3). These data were used for descriptive comparison of biomass and extracellular pigment-production trends and for selecting suitable cultivation conditions within the tested ranges. No pairwise statistical significance lettering was assigned to these descriptive fermentation-screening datasets; therefore, the error bars indicate data dispersion among replicates and should not be interpreted as direct indicators of statistical significance. For the RSM model, analysis of variance was performed in Design-Expert 13 to evaluate model significance, lack of fit, and coefficients of determination.

## 3. Results and Discussion

### 3.1. Morphological and Molecular Identification of Strain H-1

**Figure 1:**
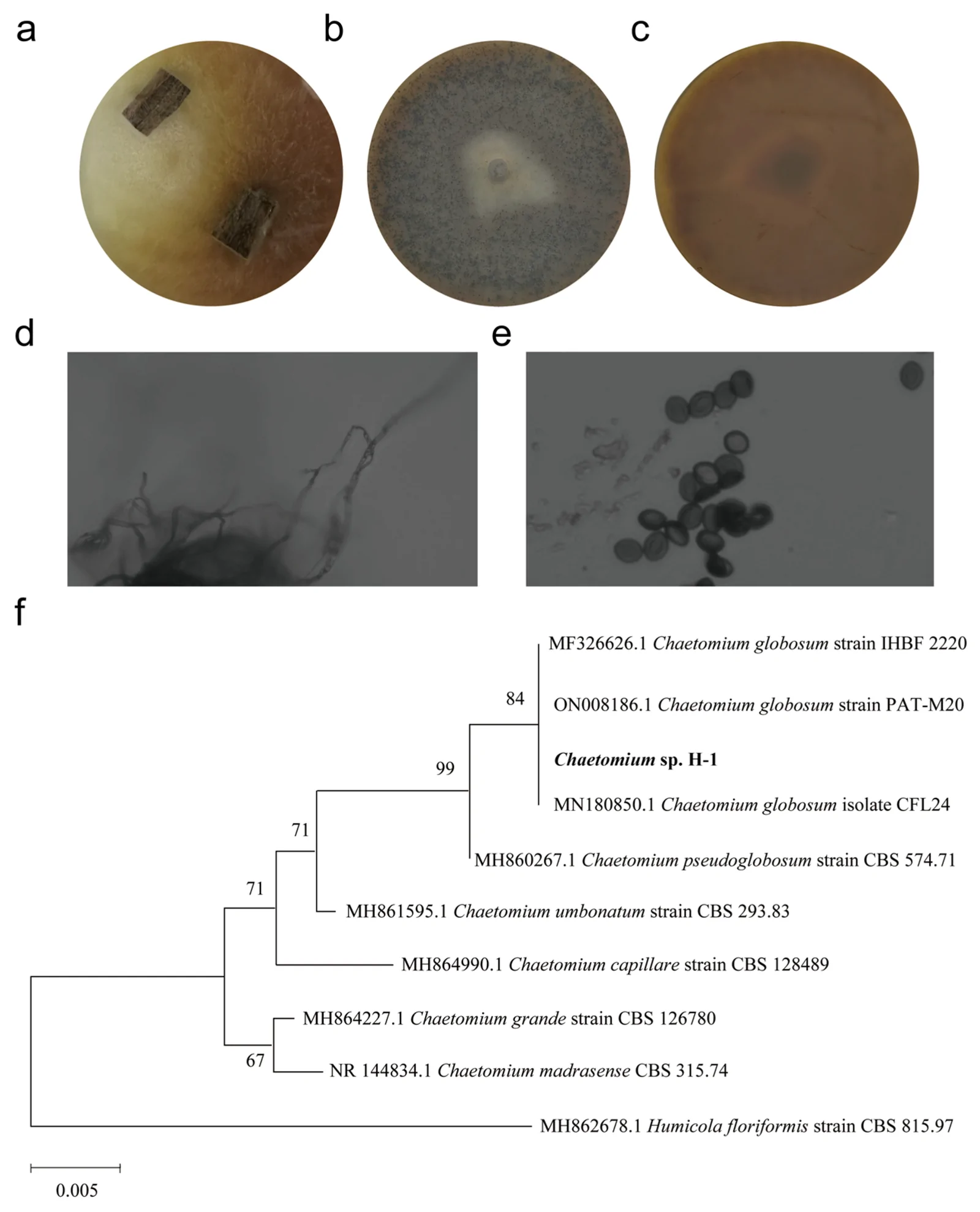
Morphological and molecular identification of strain H-1. (**a**) Isolation plate of spalted wood tissue; (**b**) front view of PDA colony; (**c**) reverse view of PDA colony; (**d**) appendage hairs of ascomata (40×); (**e**) ascospores (100×); (**f**) neighbor-joining phylogenetic tree based on ITS sequences.

After tissue isolation and consecutive purification, a fungal strain stably secreting yellow pigment, H-1, was obtained. The strain expanded from spalted wood tissue on PDA plates and gradually formed a distinct yellow coloration zone (Figure 1a). The purified strain grew rapidly on PDA, initially producing white floccose mycelia and subsequently covering the plate while the colony color changed from yellow-brown to gray-brown; the reverse side of the plate showed a uniform brown-yellow color (Figure 1b,c). Microscopic observation revealed slender appendage hairs with clear septa and mature spores that were lemon-shaped or elliptical and dark brown (Figure 1d,e), consistent with the typical morphological characteristics of *C. globosum*.

The ITS sequence of H-1 was 552 bp in length. In the phylogenetic tree, H-1 clustered with three *C. globosum* strains in the same branch, with a bootstrap support value of 84 (Figure 1f). Based on colony morphology, appendage hair and spore features, and ITS sequence relationships, strain H-1 was identified as *C. globosum*, and the sequence accession number was PQ573295 [16].

### 3.2. Stepwise Screening of Extracellular Pigment Production

Before fermentation screening, the UV-Vis spectrum of the extracellular pigment preparation was recorded to determine an appropriate monitoring wavelength. As shown in Figure S1a, the pigment solution exhibited a distinct absorption maximum at 273 nm and a gradually decreasing absorption profile extending into the visible region, which was consistent with its yellow-brown appearance. In addition, the OD_273_ value showed a strong linear relationship with pigment concentration within 0.1–1.0 g/L, with the calibration equation y = 2.0719x + 0.0277 and R^2^ = 0.999 (Figure S1b). Therefore, OD_273_ was used in this study as a relative index for extracellular pigment-containing chromophoric products during fermentation screening, rather than as a selective quantification of a single purified yellow compound.

**Figure 2:**
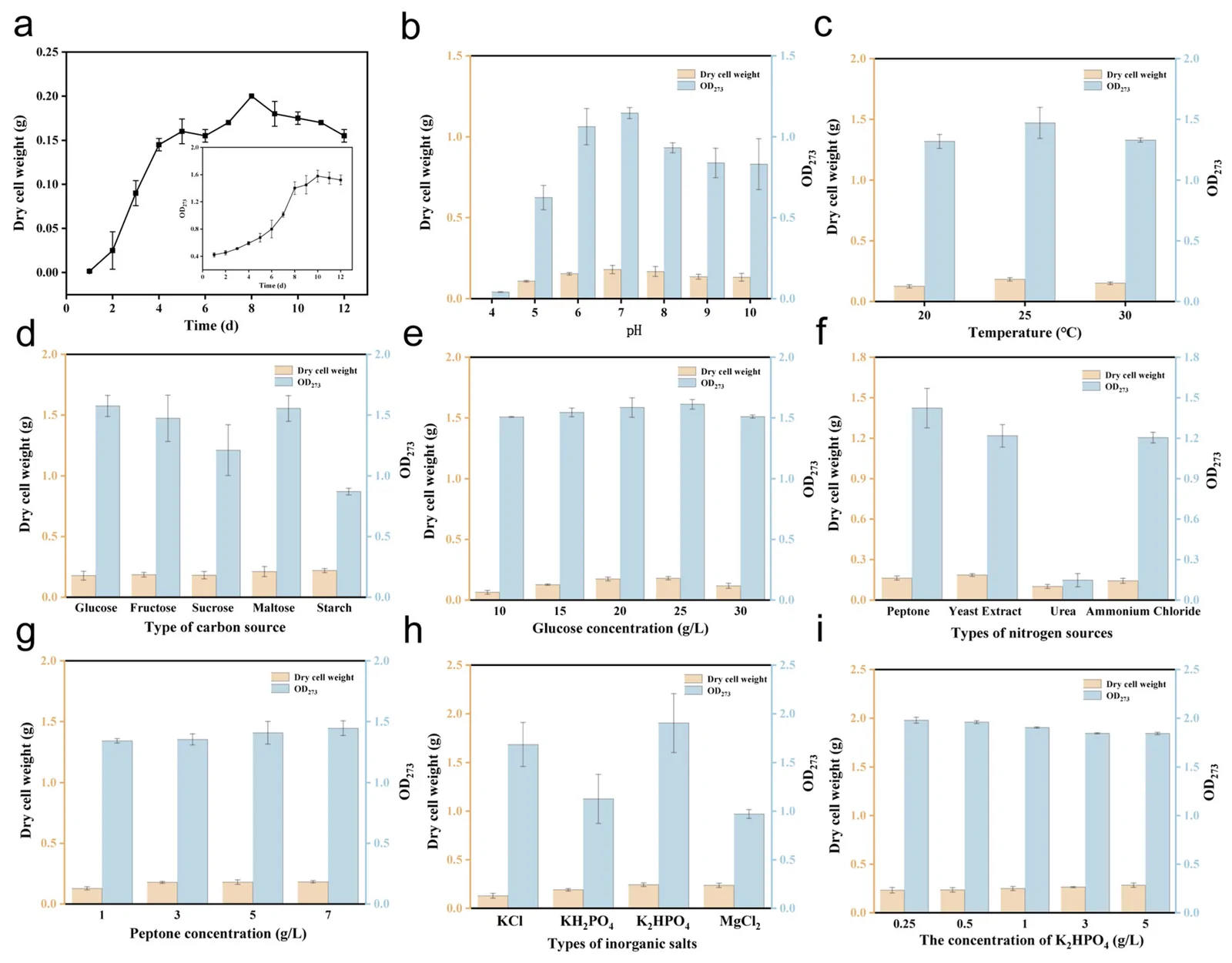
Stepwise screening of extracellular yellow pigment production by strain H-1. (**a**) Cultivation time; (**b**) initial pH, with pigment production expressed as ΔOD_273_; (**c**) cultivation temperature; (**d**,**e**) carbon source type and glucose concentration; (**f**,**g**) nitrogen source type and fish peptone concentration; (**h**,**i**) inorganic salt type and K_2_HPO_4_ concentration affecting DCW and OD_273_. Data are presented as mean ± SD (stepwise fermentation screening, n = 5; growth curve, n = 3). Error bars indicate replicate variability and do not denote pairwise statistical significance.

During liquid cultivation, the DCW of H-1 increased continuously during the first 8 d and reached approximately 0.20 g, followed by a slight decline; OD_273_ increased rapidly from days 7 to 9 and plateaued after day 10 (Figure 2a). The coordinated sequence between biomass growth and pigment accumulation indicates that extracellular yellow pigment formation mainly occurred during the late vigorous-growth phase and the stationary phase. Considering both pigment yield and cultivation period, 10 d was selected as the subsequent cultivation time.

The strain grew and produced pigment over the initial pH range of 5–10. At pH 7, DCW and ΔOD_273_ reached 0.180 ± 0.026 g and 1.146 ± 0.034, respectively (Figure 2b). Temperatures of 20–30 °C supported fungal growth, and at 25 °C, DCW and OD_273_ were 0.184 ± 0.014 g and 1.471 ± 0.128, respectively (Figure 2c). These results indicate that H-1 has broad environmental adaptability and achieves coordinated enhancement of growth and pigment production under near-neutral conditions at 25 °C [30,31].

Stepwise screening of nutrient sources further identified conditions that enhanced pigment accumulation. The glucose group produced the highest OD_273_ (1.575 ± 0.087), with an optimal concentration of 25 g/L (Figure 2d,e). Fish peptone promoted pigment production more effectively than the other nitrogen sources, and the highest pigment yield was obtained at 7 g/L (Figure 2f,g). K_2_HPO_4_ simultaneously promoted fungal growth and extracellular pigment formation, and OD_273_ reached 1.979 ± 0.030 at 0.25 g/L (Figure 2h,i). According to the calibration equation shown in Figure S1b, this OD_273_ value corresponded to approximately 0.94 ± 0.01 g/L crude lyophilized pigment-equivalent concentration. The results identify suitable conditions within the tested ranges, but further multifactorial designs, independent batch validation, and scale-up studies are needed to improve process robustness and scalability. Thus, the main composition of the pigment-producing medium was determined as 25 g/L glucose, 7 g/L fish peptone, and 0.25 g/L K_2_HPO_4_ [5,6,32]. Nevertheless, the fermentation screening used in this study was not a factorial or response-surface DoE and therefore could not quantify interaction effects among initial pH, temperature, carbon source, nitrogen source, and inorganic salt. The identified conditions should therefore be regarded as suitable conditions within the tested single-factor ranges rather than globally optimized fermentation conditions. Future work should apply factorial designs or response-surface methodology to evaluate factor interactions, verify independent batch-to-batch reproducibility, and improve the robustness and scalability of pigment production.

### 3.3. Chemical Characteristics and Antibacterial Activity

**Figure 3:**
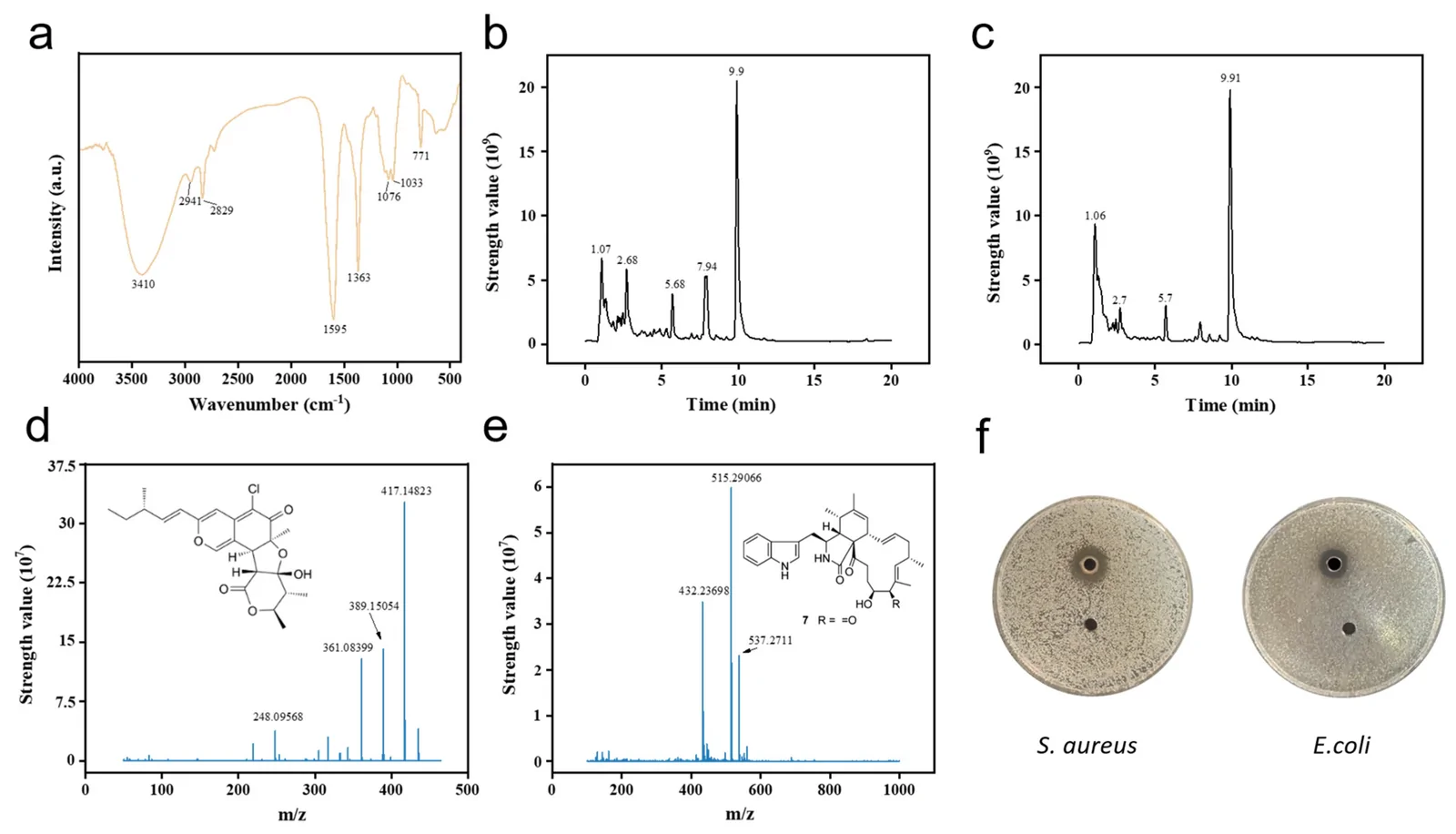
Chemical characteristics and antibacterial activity of the H-1 extracellular pigment. (**a**) FTIR spectrum; (**b**,**c**) total ion chromatograms in positive and negative ion modes; (**d**) MS^2^ spectrum and structure of Chaetomugilin D; (**e**) mass spectrum and structure of Armochaetoglobin G; (**f**) enlarged images showing the antibacterial effects of the pigment solution against *S. aureus* and *E. coli*. The agar wells used in the diffusion assay were 6 mm in diameter.

The FTIR spectrum of the pigment showed a broad and strong O-H stretching vibration band at 3410 cm^−1^, aliphatic C-H stretching vibrations at 2941–2829 cm^−1^, an aromatic skeleton vibration at 1595 cm^−1^, C-H bending at 1363 cm^−1^, C-O-C stretching vibrations at 1076 and 1033 cm^−1^, and substituted aromatic C-H out-of-plane bending at 771 cm^−1^ (Figure 3a). These features indicate that the H-1 extracellular pigment is rich in hydroxylated aromatic structures and ether-bond-containing components, providing a structural basis for aqueous dispersion, radical reactions, and fiber binding [33].

The total ion chromatograms in both positive and negative ion modes showed a dominant signal at approximately 9.9 min (Figure 3b,c). However, this peak could not be assigned confidently based on the available accurate-mass and MS/MS information; therefore, it was not identified as a specific compound in this study. Instead, the UHPLC-HRMS/MS analysis was used to annotate representative Chaetomium-related metabolites with clearer ion and fragment information. The component at retention time (RT) 7.94 min exhibited m/z 435.15641 [M + H]^+^ and 433.14104 [M − H]^−^, with fragment ions at m/z 417.14823, 389.15054, 361.08399, and 248.09568, and was putatively annotated as Chaetomugilin D (Figure 3d). The component at RT 8.90 min showed m/z 515.29066 [M + H]^+^ and 537.2711 [M + Na]^+^, together with characteristic fragments, and was putatively annotated as Armochaetoglobin G (Figure 3e). These results indicate that the extracellular pigment preparation contained Chaetomium-derived secondary metabolites, while the dominant total ion chromatogram (TIC) peak remains to be structurally elucidated in future work. Therefore, Chaetomugilin D and Armochaetoglobin G should be regarded as putatively annotated representative components rather than fully confirmed compounds or the dominant constituents of the preparation. The TIC profiles also indicate that the crude pigment preparation contained both major and minor components, with the most intense signal appearing at approximately 9.9 min. Future work will focus on preparative chromatographic separation of the dominant and annotated fractions, followed by NMR-based structure elucidation and comparison with authentic standards or isolated reference compounds when available. It should also be noted that *C. globosum* is known to produce bioactive secondary metabolites, including chaetoglobosin-type cytochalasans with reported cytotoxicity. Therefore, the present UHPLC-HRMS/MS annotation should not be interpreted as a safety assessment of the crude pigment preparation. No targeted mycotoxin screening, cytotoxicity assay, skin-irritation test, or allergenicity evaluation was performed in this study. Accordingly, the dyeing experiments should be regarded as a proof-of-concept evaluation of coloration performance, and further safety assessment is required before any skin-contact or consumer-product application. The pigment solution produced clear inhibition zones against both *S. aureus* and *E. coli*, with diameters of 14.30 ± 0.04 and 10.01 ± 0.17 mm, respectively (Figure 3f), indicating measurable inhibitory effects under the agar-well diffusion conditions. However, the agar-well diffusion assay provides only preliminary qualitative or semi-quantitative evidence of inhibitory activity. The diameter of the inhibition zone is influenced not only by antimicrobial potency, but also by the diffusion ability of the tested substances in agar, sample concentration, molecular size, and the growth characteristics of the indicator strains. Therefore, the present inhibition-zone results should not be used to directly rank the antimicrobial potency of the pigment preparation against other natural pigments. Minimum inhibitory concentration (MIC) and minimum bactericidal concentration (MBC) assays based on broth dilution would provide more quantitative and comparable information. Because only two indicator bacteria were tested, and MIC/MBC values and antibiotic positive controls were not included in this study, the antibacterial results are interpreted conservatively as preliminary inhibitory effects. Future work should determine MIC and MBC values using purified or fractionated pigment preparations, together with standard antibiotic controls [16,17,18,19].

### 3.4. Stability of the Extracellular Pigment

**Figure 4:**
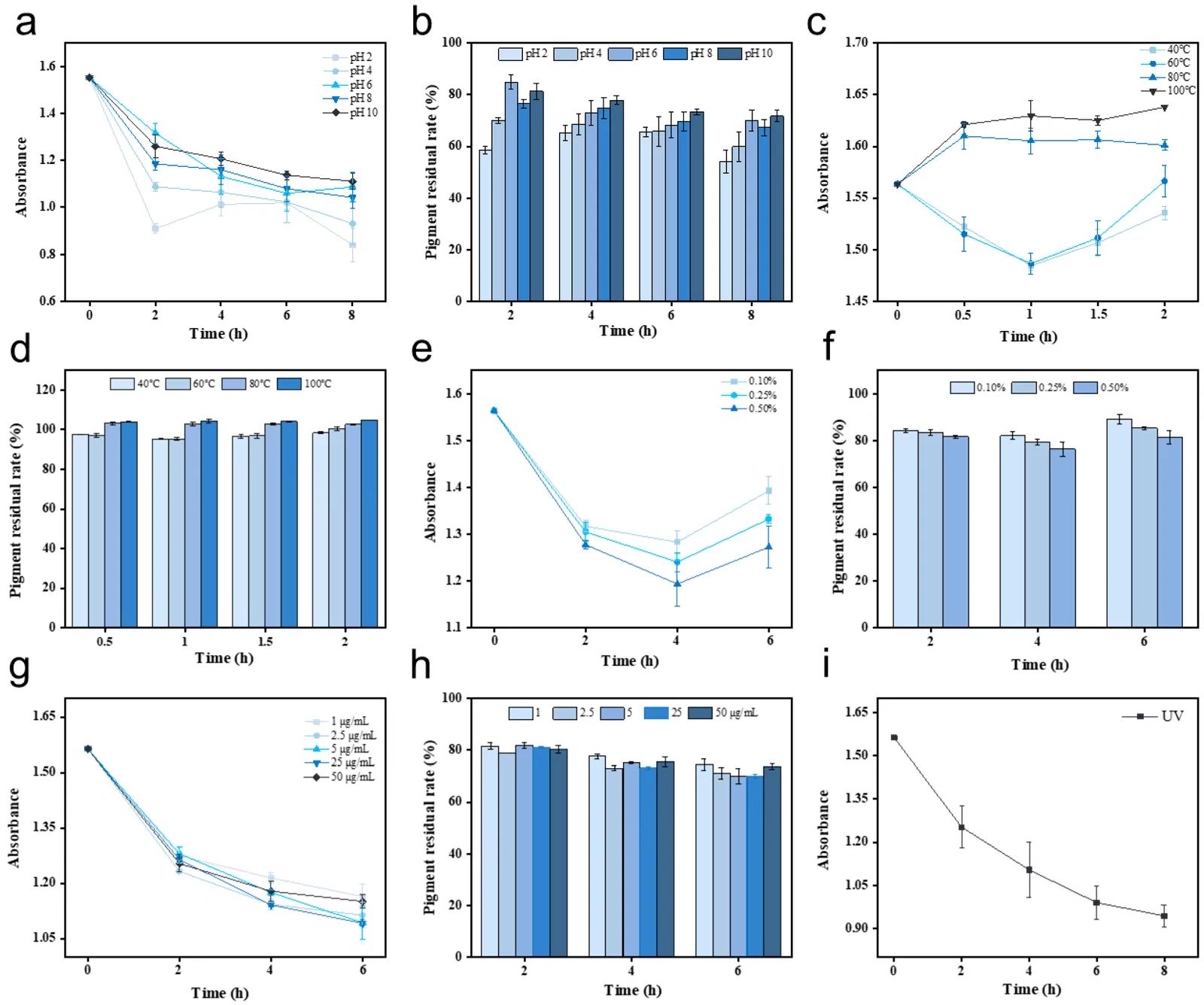
Stability of the H-1 extracellular pigment. Effects of (**a**,**b**) pH; (**c**,**d**) temperature; (**e**,**f**) H_2_O_2_; (**g**,**h**) Na_2_SO_3_; and (**i**) UV irradiation on OD_273_ or pigment residual rate. Data are presented as mean ± SD (n = 3). Pigment residual rate represents OD_273_ retention of the extracellular pigment solution after treatment and is not expressed as % *w*/*w* of biomass.

pH strongly regulated the absorbance characteristics of the pigment (Figure 4a,b). After 8 h at pH 2, the residual rate was 54.07%, whereas the residual rates under pH 6, 8, and 10 were 68.23%, 67.10%, and 71.54%, respectively, indicating that neutral to alkaline environments were more favorable for maintaining pigment color. It should be noted that the residual rate in this assay was calculated from OD_273_; therefore, the decrease under strongly acidic conditions may reflect not only pigment loss, but also acid-induced changes in the chromophoric system and absorbance profile. In the pH 2 treatment, the solution became pale golden after 8 h, and slight precipitation was observed, suggesting that protonation, aggregation/precipitation, and partial degradation of pigment-related components may have contributed to the decrease in OD_273_. During thermal treatment, OD_273_ in the 40 and 60 °C groups decreased slightly and then recovered, while the 80 and 100 °C groups increased slightly and remained stable; residual rates remained approximately 94–104% within 2 h (Figure 4c,d), indicating excellent short-term thermal stability [34,35].

Under H_2_O_2_ treatment, pigment absorbance showed a dynamic decrease followed by recovery. At 4 h under 0.50% H_2_O_2_, the residual rate was 76.33%, and after 6 h, all groups remained at approximately 81–89% (Figure 4e,f). After Na_2_SO_3_ treatment for 6 h, residual rates were 69.83–74.44%, and the trends among different concentration groups were similar (Figure 4g,h). After UV irradiation for 8 h, the residual rate was 60.32% (Figure 4i; Figure S2). Metal ions also affected pigment stability to different extents (Table S1). Li^+^ and Zn^2+^ had relatively minor effects, with residual rates of 96.54% and 96.46%, respectively. K^+^, Na^+^, Mg^2+^, Ca^2+^, and Mn^2+^ caused moderate decreases in OD_273_ but did not markedly alter the yellow-brown tone. In contrast, Pb^2+^ caused a pronounced decrease in residual rate to 52.99% with visible precipitation, while Fe^3+^ increased OD_273_ to 121.92% and changed the solution to a dark brown/black-brown tone. Cu^3+^ and Al^3+^ also induced color changes and precipitation, suggesting possible pigment-metal complexation or Aggregation. Overall, the pigment exhibited favorable short-term thermal stability and adaptability to oxidative environments, while neutral to alkaline, light-avoiding, non-reductive, and metal-ion-controlled conditions better preserved its color performance [34,35,36].

### 3.5. Antioxidant Characteristics During Fermentation

**Figure 5:**
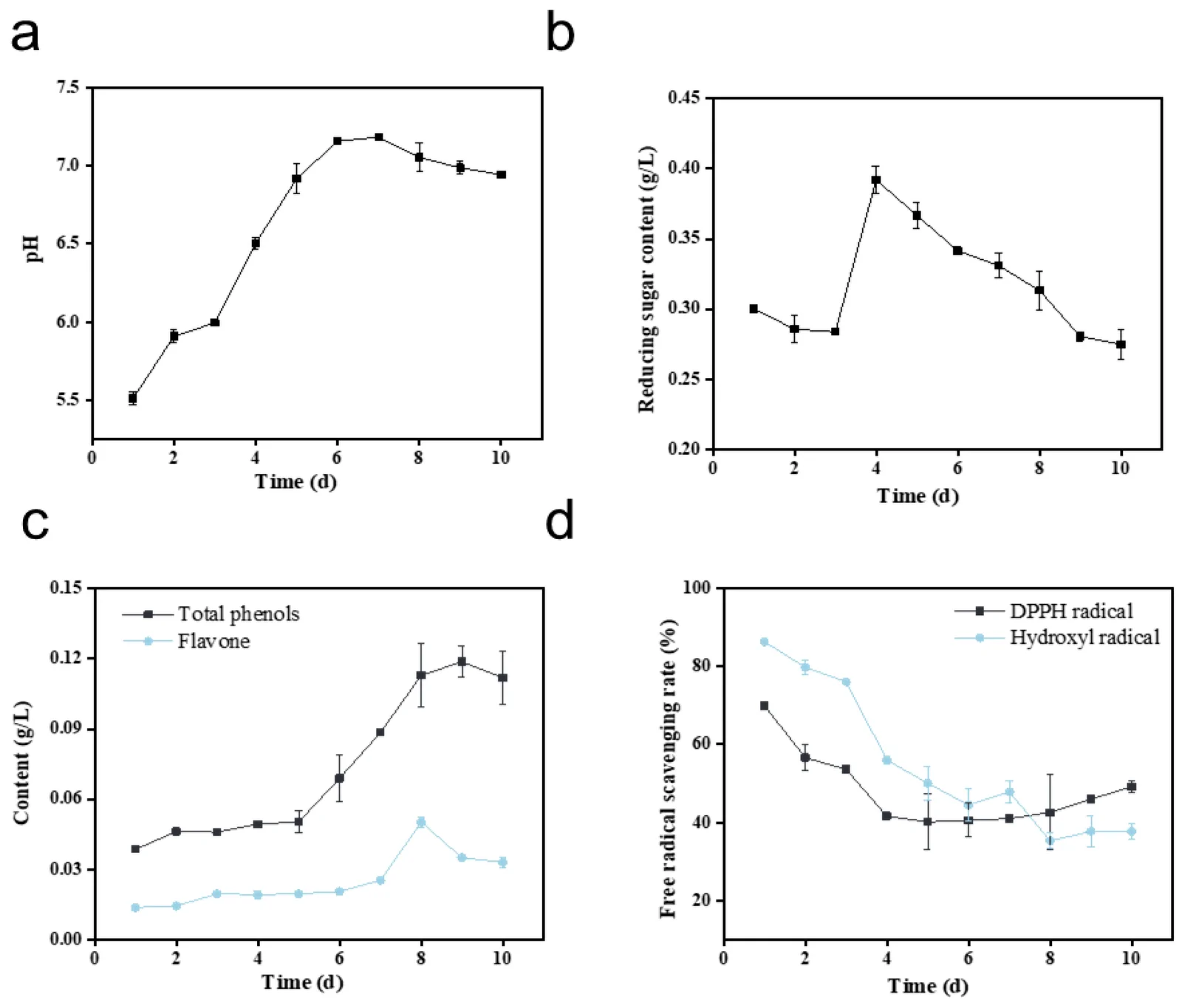
Antioxidant-related indicators of the extracellular pigment solution during liquid fermentation of H-1. (**a**) pH; (**b**) reducing sugar; (**c**) total phenol and flavonoid contents; (**d**) DPPH and hydroxyl radical-scavenging activities. Data are presented as mean ± standard deviation (n = 3).

The pH of the fermentation filtrate increased gradually from approximately 5.5 on day 1 to approximately 7.2 on day 7 and then remained stable (Figure 5a). Reducing sugar decreased slightly during the first 3 d, increased to 0.3921 g/L on day 4, and then decreased continuously with fungal growth and secondary metabolism (Figure 5b). Total phenol and flavonoid contents reached their maxima of 0.1188 g/L on day 9 and 0.0502 g/L on day 8, respectively (Figure 5c), and their accumulation stages coincided with the late growth period and concentrated extracellular pigment formation [37,38,39,40].

The pigment-containing fermentation filtrates showed hydroxyl and DPPH radical-scavenging capacities throughout the cultivation period (Figure 5d). Hydroxyl radical-scavenging activity gradually changed from approximately 86% at the early stage to 35.33% on day 8 and then recovered. DPPH radical-scavenging activity decreased from 70.00% on day 1 to 40.15% on day 5 and then recovered to approximately 49% at the late stage. These results indicate that the crude fermentation filtrates retained measurable radical-scavenging capacity throughout cultivation.

The radical-scavenging curves, together with the changes in pH, total phenols, flavonoids, and pigment accumulation, suggest that the antioxidant behavior of the H-1 fermentation system was associated with multiple extracellular metabolites rather than a single purified pigment compound [39,41]. The changes in total phenols and flavonoids were not fully consistent with the radical-scavenging curves, indicating that other extracellular metabolites and pH-related changes may also have contributed to the observed antioxidant activity. Therefore, the present DPPH results should be interpreted as preliminary evidence of radical-scavenging capacity in crude fermentation filtrates rather than as a quantitative benchmark of antioxidant potency. Because no concentration-response assay, purified pigment fraction, or standard antioxidant control was included, IC50 values and Trolox-equivalent antioxidant capacity could not be obtained, and the antioxidant capacity of the pigment preparation cannot be directly ranked against other natural dyes or standard antioxidants. Future work should evaluate purified or fractionated pigment samples using standardized DPPH/2,2’-azino-bis(3-ethylbenzothiazoline-6-sulfonic acid) (ABTS) assays, IC50 determination, and Trolox-equivalent antioxidant capacity analysis.

### 3.6. Wool Dyeing Performance and Dye-Bath Reuse

**Figure 6:**
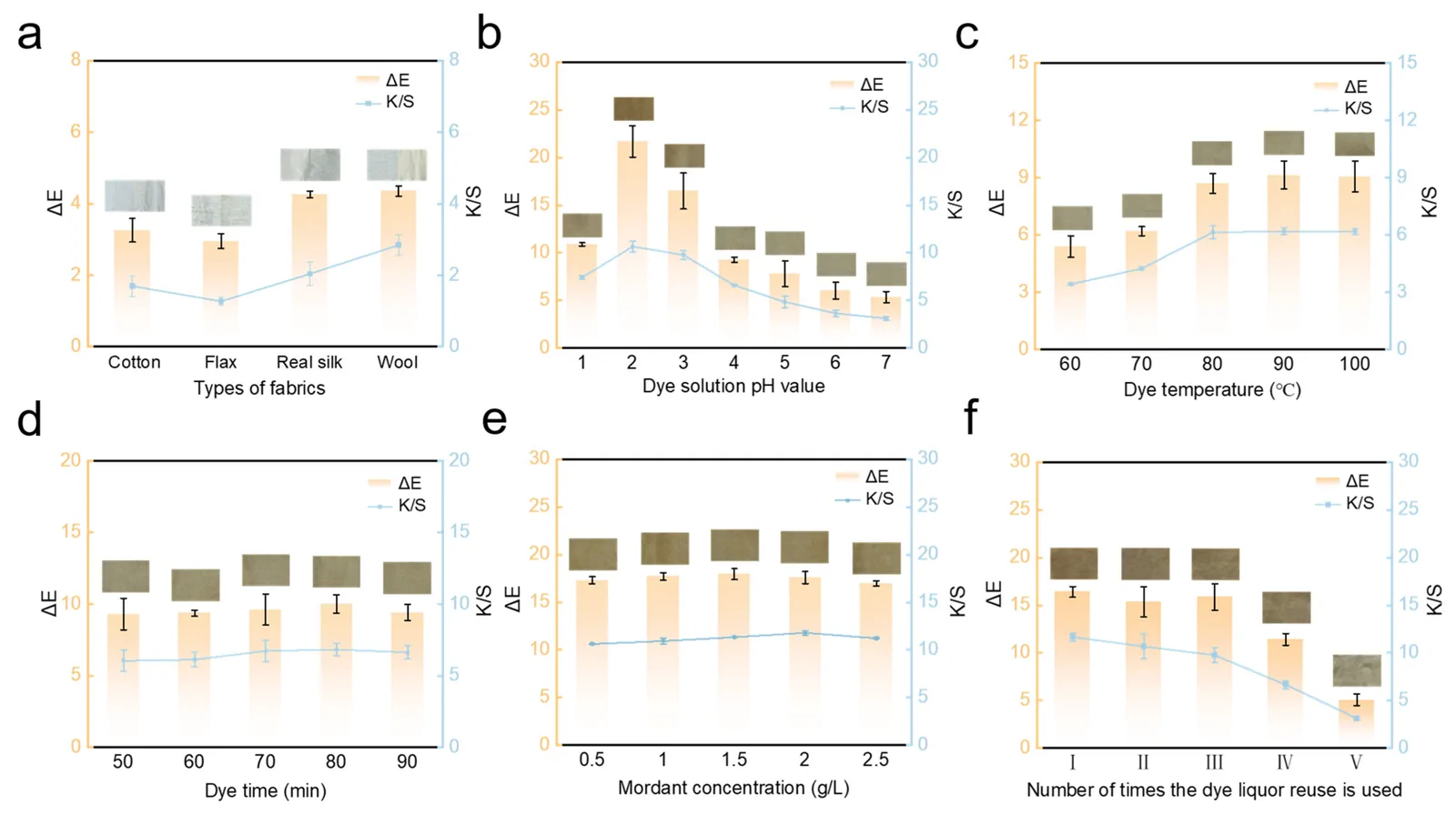
Wool dyeing process using the H-1 extracellular pigment. Effects of (**a**) fabric type; (**b**) dye-bath pH; (**c**) dyeing temperature; (**d**) dyeing time; (**e**) potassium aluminum sulfate concentration; and (**f**) dye-bath reuse cycle on ΔE and K/S. Data are presented as mean ± standard deviation (n = 3).

All four fabrics developed yellow-brown tones under the same dyeing conditions, with wool showing the highest ΔE and K/S values, followed by silk, whereas cotton and linen showed lower values (Figure 6a; Table S2). The amino, carboxyl, and amide groups in protein fibers provide abundant binding sites for pigment molecules, and the scale layer of wool opens under thermal treatment to facilitate pigment diffusion into the fiber interior. Consequently, wool exhibited higher dye affinity [22,24].

At dye-bath pH 2, wool achieved the highest ΔE and K/S values (Figure 6b; Table S3). The different outcomes of the pH-stability and dyeing experiments arise from their different evaluation systems. The stability assay examined the pigment solution alone during 8 h of acid exposure, whereas dyeing was a shorter adsorption/fixation process involving wool fibers and potassium aluminum sulfate. Under acidic dye-bath conditions close to pH 2, amino groups in wool are protonated, which can enhance electrostatic interactions with anionic or oxygen-containing pigment components. In addition, Al^3+^ can promote coordination bridging between pigment molecules and wool functional groups. Thus, although strong acidity is unfavorable for long-term solution stability, a moderately acidic dye bath can promote pigment adsorption and fixation on wool, resulting in higher K/S values. K/S increased as the dyeing temperature rose to 90 °C and reached the optimum color depth at 80 min (Figure 6c,d; Tables S4 and S5). Simultaneous addition of potassium aluminum sulfate significantly enhanced pigment fixation, and K/S reached 11.79 at 2 g/L (Figure 6e; Table S6). Acidic conditions enhanced electrostatic interactions between wool fibers and anionic groups in the pigment, while Al^3+^ further strengthened pigment-fiber binding through coordination bridging, producing a fuller yellow-brown shade [24,25].

After three consecutive dye-bath reuse cycles, ΔE and K/S decreased by 3.32% and 16.28%, respectively, and the fifth dyeing cycle still produced a distinct yellow tone (Figure 6f; Table S7). Under the optimized dyeing conditions, the dyed wool showed washing color-change fastness of grade 4, cotton staining fastness of grade 4, wool staining fastness of grade 3–4, dry rubbing fastness of grade 4, and wet rubbing fastness of grade 3–4 (Table S8). During dye-bath reuse, the washing and rubbing fastness ratings of all dyed samples remained above grade 3 (Table S9). These results indicate that the pigment-mordant-wool system provided acceptable resistance to washing and rubbing under the GB/T test procedures used in this study. However, because lightfastness and internationally harmonized AATCC/ISO repeated laundering tests were not included, the present fastness results should be considered an initial evaluation of washing and rubbing durability rather than a complete textile durability assessment. Further work should evaluate lightfastness and repeated wash durability using recognized international standards such as AATCC or ISO methods. During dye-bath reuse, the OD_273_ and Al^3+^ values of the residual baths decreased gradually with the number of cycles (Figure S3; Table S10). Because the bath was replenished with water after each cycle to restore the liquor ratio, these values represent apparent residual concentrations rather than pure depletion by fiber uptake alone. The apparent decrease in Al^3+^ concentration was calculated relative to the residual bath after the first cycle: from 18.13 mg/L after cycle I to 0.83 mg/L after cycle V, corresponding to [(18.13 − 0.83)/18.13] × 100 = 95.42%. Similarly, OD_273_ decreased from 0.3794 after cycle I to 0.1505 after cycle V, corresponding to an apparent decrease of 60.33%. The different decreases in K/S and ΔE during the first three cycles can be attributed to the different meanings of the two color indices. K/S is calculated from reflectance at the selected absorption wavelength and is more sensitive to color depth, whereas ΔE reflects the overall distance in L*a*b* color space relative to the undyed fabric. During cycles I-III, the L* and a* values changed moderately, while b* remained positive and even increased in cycle III, indicating that the yellow tone was largely retained. Therefore, the overall color difference changed only slightly, whereas the reflectance-based color strength decreased more noticeably. This recycling process achieved stepwise utilization of pigment and mordant and produced stable color gradations from deep to light shades [26]. For risk mitigation in future textile applications, pigment preparations should be purified or fractionated to remove undesired secondary metabolites, followed by targeted mycotoxin profiling, cytotoxicity testing, skin-irritation/sensitization assessment, and evaluation of residual extractables on dyed fabrics. Until such data are available, the crude pigment preparation should be handled as a fungal metabolite mixture for laboratory research rather than as a confirmed safe colorant for consumer products.

### 3.7. Response Surface Optimization and Model Validation

The quadratic regression equation for K/S established by the Box–Behnken experiment was as follows: Y = 11.37 + 0.8225A + 0.0833B + 0.0583C + 0.0958D − 0.0175AB + 0.0375AC − 0.1225AD + 0.0775BC + 0.0550BD − 0.0700CD − 1.15A^2^ − 0.3125B^2^ − 0.6975C^2^ − 0.3487D^2^.

**Figure 7:**
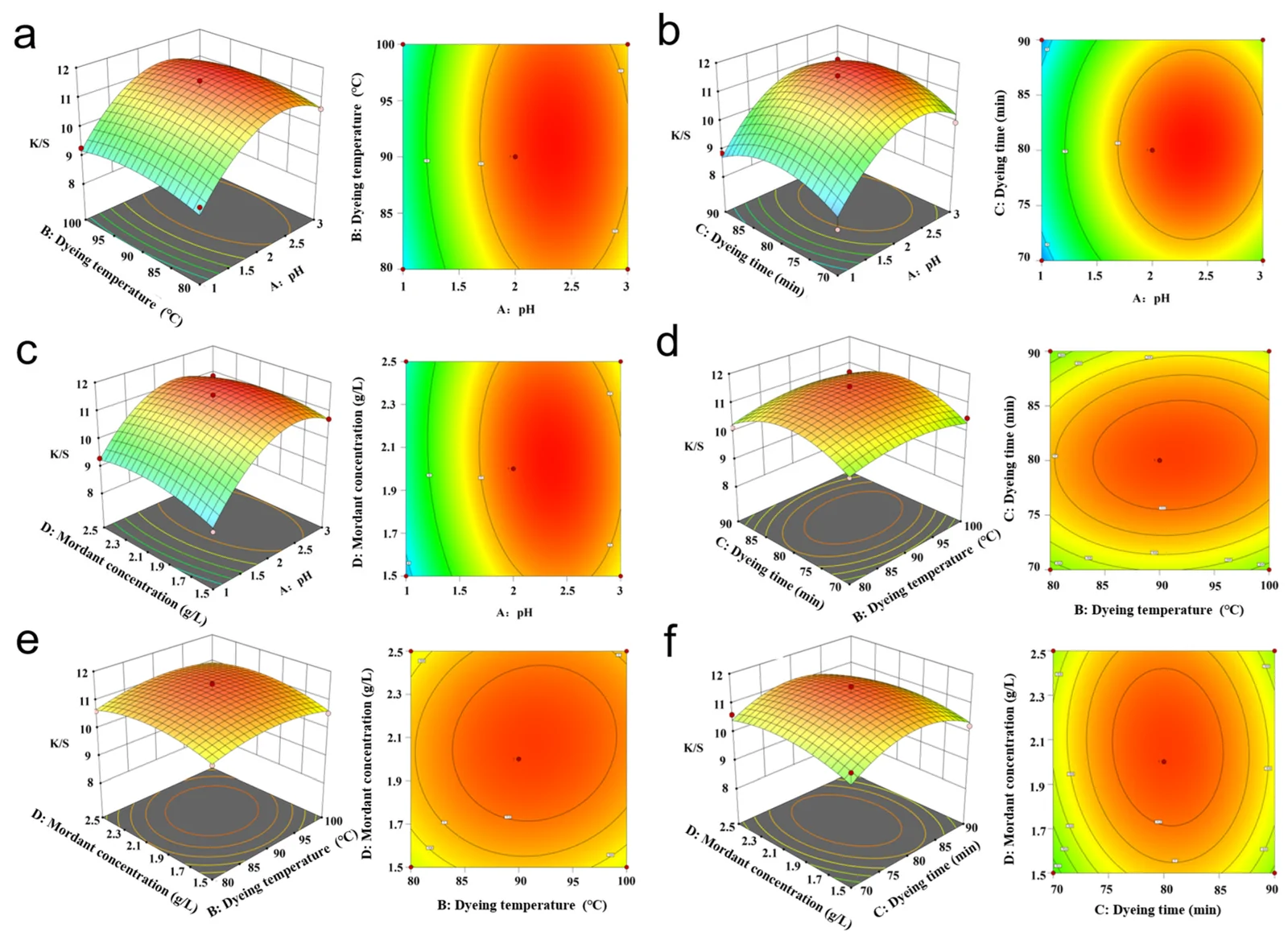
Response surface and contour plots showing the effects of dyeing factors on K/S. (**a**) pH-temperature; (**b**) pH-time; (**c**) pH-mordant concentration; (**d**) temperature-time; (**e**) temperature-mordant concentration; (**f**) time-mordant concentration.

The overall quadratic model was highly significant (F = 14.36, *p* < 0.0001), and the lack of fit was not significant (*p* = 0.3130), indicating that the model adequately described the experimental domain (Tables S11 and S12). The R^2^ and adjusted R^2^ values were 0.9437 and 0.8780, respectively. Although the difference between R^2^ and adjusted R^2^ suggests that some terms contributed less strongly to the response, the adjusted R^2^ value remained acceptable for a four-factor natural-dyeing optimization model. The linear terms B, C, and D were not statistically significant individually; however, they were retained in the regression equation to preserve the hierarchy of the full quadratic response-surface model, because the corresponding quadratic terms B^2^, C^2^, and D^2^ were significant. Therefore, the model was used for interpolation and process optimization within the tested factor ranges rather than for mechanistic interpretation of each linear coefficient. The linear and quadratic terms of dye-bath pH were highly significant, and the quadratic terms of temperature, time, and mordant concentration were also significant. The response surfaces in Figure 7 were continuous and convex, with the maximum response region concentrated at pH 2.0–2.5, approximately 90 °C, approximately 80 min, and approximately 2.0 g/L mordant; among the factors, pH exerted the most pronounced regulatory effect on K/S.

The predicted maximum K/S value was 11.5229, corresponding to pH 2.3527, 91.3845 °C, 80.5578 min, and 2.0404 g/L potassium aluminum sulfate. Using the practical validation conditions of pH 2.35, 91 °C, 80 min, and 2.04 g/L, the mean validation K/S value from three parallel experiments was 11.65, with a relative deviation of 1.09% from the predicted value. This result verified the predictive accuracy of the model for wool color depth. RSM has been widely used to optimize natural dyeing systems because dye-bath pH, temperature, dyeing time, dye concentration, and mordant dosage can jointly affect color strength and fastness. For example, Haji and Rahimi [42] optimized wool dyeing with Berberis thunbergii DC leaves using RSM and showed that dye concentration, dye-bath pH, temperature, and mordant concentration affected K/S. Patil et al. [43] also used a Box–Behnken design to optimize pashmina wool dyeing with Butea monosperma flower extract and validated the model by comparing predicted and experimental K/S values. In fungal-pigment dyeing, Hernández et al. [44] reported that extracellular dyes from wood-inhabiting fungi, including Talaromyces australis and Penicillium murcianum, could dye wool with acceptable washing fastness under textile-relevant conditions. Compared with these studies, the novelty of the present work lies not in the use of RSM alone, but in applying a Box–Behnken dyeing model to a *Chaetomium globosum*-derived extracellular yellow pigment system and integrating substrate screening, simultaneous aluminum mordanting, model validation, color-fastness evaluation, and dye-bath reuse. The optimized K/S value of 11.65 under pH 2.35, 91 °C, 80 min, and 2.04 g/L potassium aluminum sulfate indicates that this crude extracellular pigment preparation can produce a relatively deep yellow-brown shade on wool within the tested domain, while the dye-bath reuse results further demonstrate stepwise utilization of both pigment and mordant.

## 4. Conclusions

This study systematically developed a production and application pathway for extracellular yellow pigments from *C. globosum* H-1. Morphological and ITS sequence analyses identified the strain as *C. globosum*. Stepwise single-factor screening indicated that cultivation at an initial pH of 7 and 25 °C for 10 d using 25 g/L glucose, 7 g/L fish peptone, and 0.25 g/L K_2_HPO_4_ was suitable for fungal growth and extracellular pigment accumulation within the tested ranges. FTIR and UHPLC-HRMS/MS revealed hydroxylated aromatic structural features in the crude pigment preparation and allowed putative annotation of representative Chaetomium-related components, including Chaetomugilin D and Armochaetoglobin G, while the dominant TIC peak and individual component purity require further structural confirmation. The pigment maintained favorable stability under neutral to alkaline environments and short-term thermal treatment, while also showing preliminary inhibitory effects against *S. aureus* and *E. coli* and measurable hydroxyl and DPPH radical-scavenging capacities in crude fermentation filtrates. The extracellular yellow pigment showed strong affinity toward protein fibers, and wool dyeing performance was markedly superior to that of cotton, linen, and silk. Response surface optimization identified dyeing conditions of pH 2.35, 91 °C, 80 min, and 2.04 g/L potassium aluminum sulfate, under which the validation K/S value reached 11.65. After three dye-bath reuse cycles, high color depth and fastness ratings above grade 3 were retained, and the apparent residual Al^3+^ concentration decreased to 0.83 mg/L after the fifth cycle. This work establishes an integrated technical system encompassing fermentation enhancement, component analysis, functional evaluation, dyeing optimization, and dye-bath recycling, providing a systematic basis for further development of microbial extracellular yellow pigments as natural textile colorants, provided that future purification, mycotoxin screening, cytotoxicity, and skin-contact safety evaluations are completed.

## Data Availability

The original contributions presented in this study are included in the article/supplementary material. Further inquiries can be directed to the corresponding author.

## Supplementary Materials

The following supporting information can be downloaded at: https://www.mdpi.com/article/10.3390/microorganisms14081818/s1, Figure S1: (a) UV-Vis absorption spectrum of the extracellular pigment and (b) calibration curve between pigment concentration and OD273; Figure S2: UV stability of the pigment: pigment residual rate; Figure S3: Pigment concentration during dye-bath reuse; Table S1: Effects of different kinds of metal ions on pigment stability; Table S2: Color differences and dyeing effects of different fabric types; Table S3: Chromatic values and dyeing effects of wool dyed at different dye-bath pH values; Table S4: Chromatic values and dyeing effects of wool dyed at different dyeing temperatures; Table S5: Chromatic values and dyeing effects of wool dyed for different dyeing times; Table S6: Chromatic values and dyeing effects of wool dyed with different mordant concentrations; Table S7: Chromatic values and dyeing effects of wool during dye-bath reuse; Table S8: Color fastness of wool fabrics under optimized dyeing process; Table S9: Color fastness of wool fabrics during dye-bath reuse; Table S10: Al3+ concentration in dye baths after dye-bath reuse; Table S11: Box-Behnken experimental design and K/S response values; Table S12: Analysis of variance for the quadratic regression model of K/S.
