# Supplementary material for: Extracellular Yellow Pigments Produced by Chaetomium globosum H-1 Isolated from Spalted Wood: Fermentation Regulation, Multidimensional Characterization, and Sustainable Wool Dyeing
Source: Microorganisms. 2026 Aug 18;14(8):1818. doi: 10.3390/microorganisms14081818 (PMC13515149; doi:10.3390/microorganisms14081818)
Supplement: Supplementary file 1 [file microorganisms-14-01818-s001.zip › microorganisms-4461379-supplementary.pdf]

Supplementary Materials

Extracellular Yellow Pigments Produced by *Chaetomium globosum* H-1 Isolated from Spalted Wood: Fermentation Regulation, Multidimensional Characterization, and Sustainable Wool Dyeing

Boxi Chen [a], Mengqi Wu [a], Jianping Sun [a, *]

[a] College of Civil Engineering and Architecture, Guangxi University, Nanning, Guangxi, 530004, China

[*] Email of the corresponding author: jpsun@gxu.edu.cn

The Supplementary Materials provide additional data supporting reproducibility, including the UV-Vis full-wavelength scan and concentration-absorbance calibration curve (Figure S1), UV-stability data for pigment residual rate (Figure S2), changes in pigment concentration during dye-bath reuse (Figure S3), metal-ion stability data (Table S1), tabulated chromatic and K/S data for fabric type, dye-bath pH, dyeing temperature, dyeing time, mordant concentration, and dye-bath reuse (Tables S2-S7), color-fastness ratings under the optimized dyeing process and during dye-bath reuse (Tables S8-S9), residual Al^{3+} concentrations in reused dye baths (Table S10), and the Box-Behnken design matrix and analysis of variance for the RSM model (Tables S11-S12).

Table S1. Effects of different kinds of metal ions on pigment stability

Ion	Initial OD_{273}	OD_{273} after treatment	Residual rate/%	Visual observation	
control group	/	/	/	Dark brown	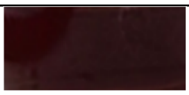
K^{+}	2.427	2.115 ± 0.054	87.17	Lighter	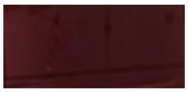
Na^{+}	2.427	2.147 ± 0.124	88.48	Lighter	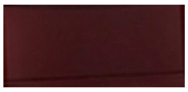
Li^{+}	2.427	2.343 ± 0.195	96.54	Dark brown	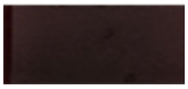
Mg^{2+}	2.427	2.121 ± 0.045	87.39	Lighter	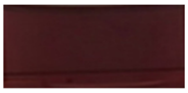
Ca^{2+}	2.427	2.050 ± 0.056	84.48	Lighter	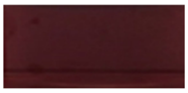
Mn^{2+}	2.427	2.157 ± 0.054	88.87	Lighter	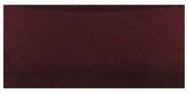
Zn^{2+}	2.427	2.341 ± 0.271	96.46	Dark brown	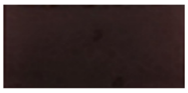
Cu^{2+}	2.427	2.090 ± 0.071	86.11	Dark reddish brown, precipitate	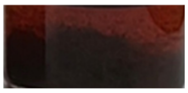
Pb^{2+}	2.427	1.286 ± 0.356	52.99	Orange-red, precipitate	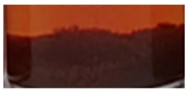
Fe^{3+}	2.427	2.959 ± 0.016	121.92	Black-brown, slight precipitate.	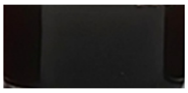
Al^{3+}	2.427	2.214 ± 0.242	91.24	Dark red, precipitate	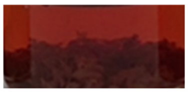

Table S2. Color differences and dyeing effects of different fabric types

Fabric type	ΔL*	Δa*	Δb*	Dyeing effect
**Cotton**	-1.78 ± 0.29	0.10 ± 0.09	2.72 ± 0.24	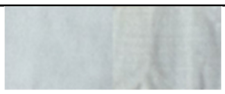
**Linen**	-1.26 ± 0.10	0.27 ± 0.04	2.65 ± 0.25	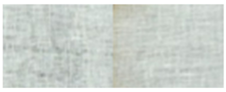
**Silk**	-2.04 ± 0.33	0.37 ± 0.17	3.71 ± 0.71	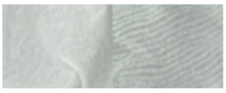
**Wool**	-2.74 ± 0.29	0.65 ± 0.15	3.32 ± 0.50	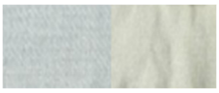

Table S3. Chromatic values and dyeing effects of wool dyed at different dye-bath pH values

Factor/level		L*	a*	b*	Dyeing effect
	1	81.89 ± 0.62	2.64 ± 0.56	15.41 ± 0.09	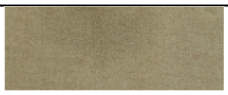
	2	72.83 ± 1.29	5.15 ± 0.54	21.44 ± 0.99	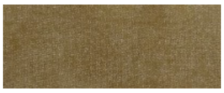
	3	77.03 ± 1.67	2.92 ± 0.54	19.05 ± 0.84	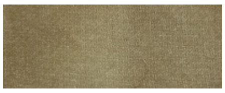
pH	4	86.48 ± 5.76	0.66 ± 0.14	14.90 ± 0.22	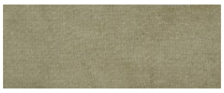
	5	84.48 ± 0.62	0.13 ± 0.19	14.83 ± 1.21	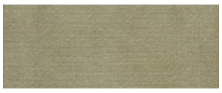
	6	85.66 ± 0.38	-0.14 ± 0.14	13.55 ± 0.84	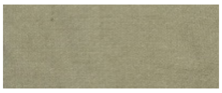
	7	86.18 ± 0.22	-0.32 ± 0.14	13.08 ± 0.58	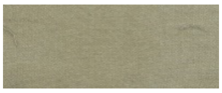

Table S4. Chromatic values and dyeing effects of wool dyed at different dyeing temperatures

**Factor/level**		L^*	a^*	b^*	**Dyeing effect**
	60	86.21 ± 0.06	-0.46 ± 0.06	12.56 ± 0.66	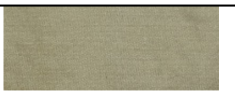
	70	85.41 ± 0.08	-0.16 ± 0.06	12.93 ± 0.24	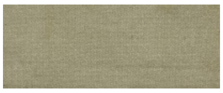
**Temperature/°C**	80	86.85 ± 5.44	0.50 ± 0.10	14.45 ± 0.51	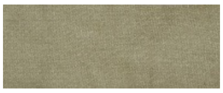
	90	83.43 ± 0.18	0.44 ± 0.09	14.99 ± 0.83	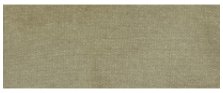
	100	83.07 ± 0.71	0.67 ± 0.31	14.50 ± 0.49	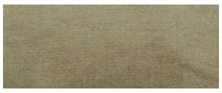

Table S5. Chromatic values and dyeing effects of wool dyed for different dyeing times

**Factor/level**		L^*	a^*	b^*	**Dyeing effect**
	50	83.23 ± 1.01	0.47 ± 0.09	15.04 ± 0.62	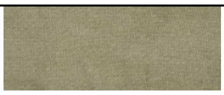
	60	83.11 ± 0.14	0.68 ± 0.11	14.99 ± 0.15	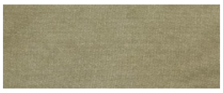
**Time/min**	70	82.87 ± 0.77	0.77 ± 0.32	15.08 ± 0.72	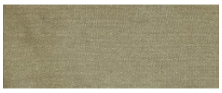
	80	82.78 ± 0.48	0.87 ± 0.18	15.51 ± 0.53	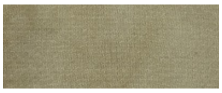
	90	82.98 ± 0.48	0.87 ± 0.24	14.86 ± 0.31	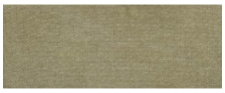

Table S6. Chromatic values and dyeing effects of wool dyed with different mordant concentrations

**Factor/level**		L^*	a^*	b^*	**Dyeing effect**
**Mordant concentration (g/L)**	**0.5**	78.63 ± 0.06	2.92 ± 0.23	21.02 ± 0.38	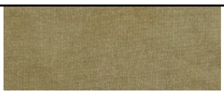
	**1**	78.28 ± 0.33	3.28 ± 0.09	21.17 ± 0.20	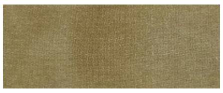
	**1.5**	77.90 ± 0.06	3.32 ± 0.08	21.14 ± 0.79	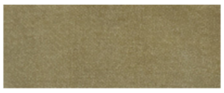
	**2**	77.33 ± 0.27	3.53 ± 0.04	19.98 ± 0.65	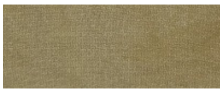
	**2.5**	77.99 ± 0.06	3.24 ± 0.18	19.84 ± 0.32	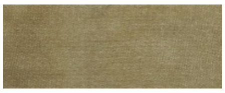

Table S7. Chromatic values and dyeing effects of wool during dye-bath reuse

**Factor/level**		L^*	a^*	b^*	**Dyeing effect**
**Reuse cycle**	**I**	76.93 ± 0.52	3.60 ± 0.24	18.57 ± 0.50	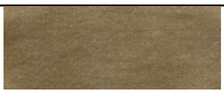
	**II**	78.01 ± 1.50	3.33 ± 0.38	18.22 ± 0.90	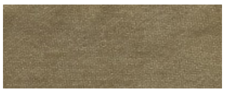
	**III**	78.96 ± 0.87	2.95 ± 0.49	20.08 ± 1.41	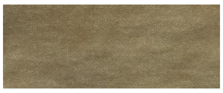
	**IV**	82.32 ± 0.44	1.63 ± 0.13	17.35 ± 0.82	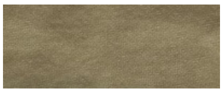
	**V**	85.99 ± 0.19	-0.12 ± 0.02	12.47 ± 0.69	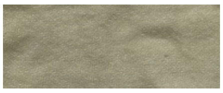

Table S8. Color fastness of wool fabrics under optimized dyeing process

**Sample**	**Fastness to soaping**			**Color fastness to rubbing**	
	**Washing color change**	**Cotton staining**	**Wool staining**	**Dry rubbing**	**Wet rubbing**
Optimized dyed wool	4	4	3-4	4	3-4

Table S9. Color fastness of wool fabrics during dye-bath reuse

Reuse cycle	Washing fastness			Rubbing fastness	
	Color change	Cotton staining	Wool staining	Dry rubbing	Wet rubbing
**I**	4-5	4	3-4	4	3-4
**II**	4	4	3-4	4	3-4
**III**	4	4	3-4	4	3-4
**IV**	4-5	4	4-5	4	4
**V**	5	5	5	5	4-5

Table S10. Al^{3+} concentration in dye baths after dye-bath reuse

Reuse cycle	Al^{3+} concentration (mg/L)
**I**	18.13
**II**	17.77
**III**	13.57
**IV**	2.24
**V**	0.83

Table S11. Box-Behnken experimental design and K/S response values

Run No.	Factor				K/S
	pH	Temperature/°C	Time/min	Mordant concentration (g/L)	
**1**	0	1	0	1	10.85
**2**	0	0	-1	1	10.62
**3**	1	0	-1	0	9.96
**4**	1	0	1	0	10.73
**5**	-1	1	0	0	9.28
**6**	0	0	1	1	10.10
**7**	1	1	0	0	10.60
**8**	0	-1	0	1	10.62
**9**	0	0	-1	-1	10.47
**10**	0	0	0	0	11.20
**11**	1	0	0	-1	10.73
**12**	1	0	0	1	10.86
**13**	0	-1	0	-1	10.54
**14**	0	1	1	0	10.67
**15**	-1	0	-1	0	8.25
**16**	-1	0	0	-1	8.69
**17**	0	-1	-1	0	10.26
**18**	-1	0	0	1	9.31
**19**	-1	-1	0	0	9.24
**20**	0	0	1	-1	10.23
**21**	1	-1	0	0	10.63
**22**	0	-1	1	0	10.14
**23**	-1	0	1	0	8.87
**24**	0	1	0	-1	10.55
**25**	0	1	-1	0	10.48
**26**	0	0	0	0	11.33
**27**	0	0	0	0	11.57

Table S12. Analysis of variance for the quadratic regression model of K/S

Source	Sum of squares	df	Mean square	F value	P value	Significance
**Model**	16.32	14	1.17	14.36	< 0.0001	**
**A-pH**	8.12	1	8.12	99.99	< 0.0001	**
**B-Temperature**	0.0833	1	0.0833	1.03	0.3310	/
**C-Time**	0.0408	1	0.0408	0.5030	0.4918	/
**D-Mordant concentration**	0.1102	1	0.1102	1.36	0.2666	/
**AB**	0.0012	1	0.0012	0.0151	0.9043	/
**AC**	0.0056	1	0.0056	0.0693	0.7968	/
**AD**	0.0600	1	0.0600	0.7394	0.4067	/
**BC**	0.0240	1	0.0240	0.2959	0.5964	/
**BD**	0.0121	1	0.0121	0.1490	0.7062	/
**CD**	0.0196	1	0.0196	0.2414	0.6320	/
**A^2**	7.07	1	7.07	87.07	< 0.0001	**
**B^2**	0.5208	1	0.5208	6.42	0.0263	*
**C^2**	2.59	1	2.59	31.96	0.0001	**
**D^2**	0.6487	1	0.6487	7.99	0.0153	*
**Residual**	0.9742	12	0.0812	/	/	/
**Lack of fit**	0.9038	10	0.0904	2.57	0.3130	/
**Pure error**	0.0705	2	0.0352	/	/	/
**Total**	17.30	26	/	/	/	/
**R^2 = 0.9437; R^2adj = 0.8780**						

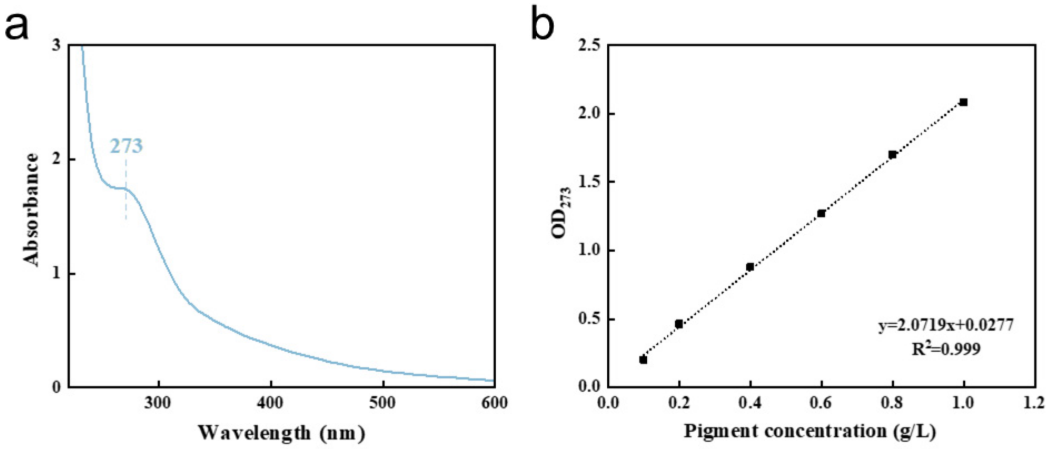

Figure S1. (a) UV-Vis absorption spectrum of the extracellular pigment and (b) calibration curve between pigment concentration and OD_{273}.

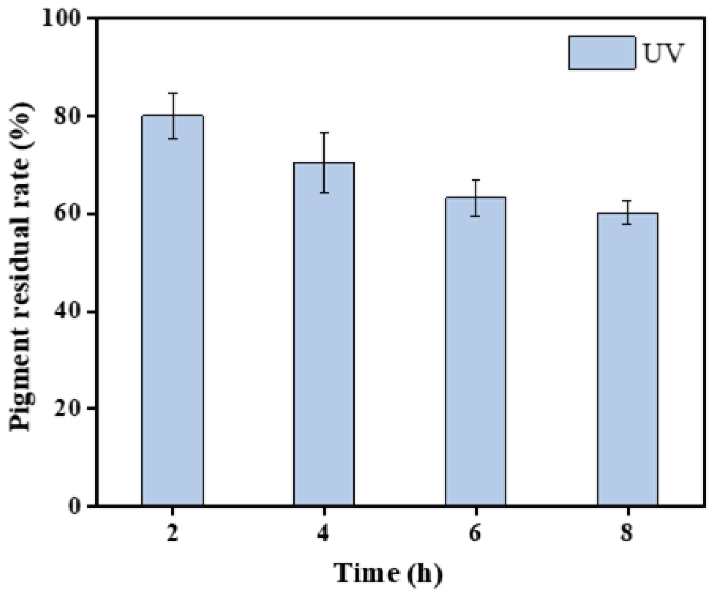

Figure S2. UV stability of the pigment: pigment residual rate

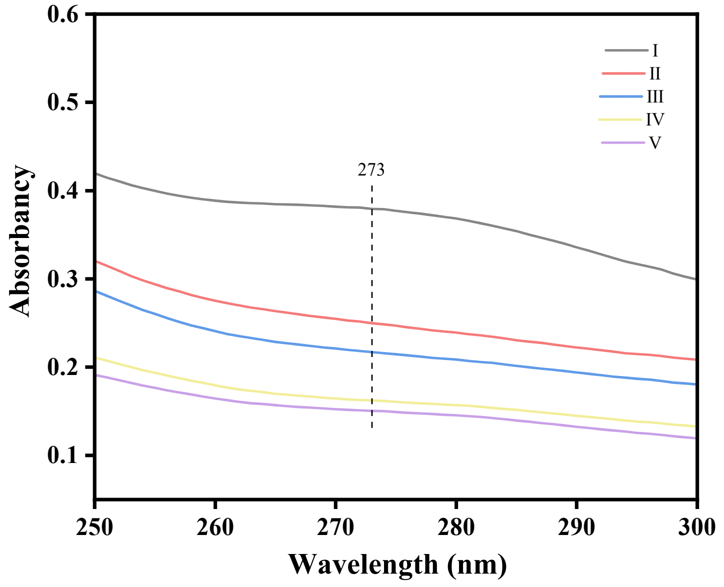

Figure S3. Pigment concentration during dye-bath reuse
